# Ovulation-Anchored Evaluation of IMU-Derived Activity and Posture-Related Behavioral Changes Across Natural Estrus Phases in Dairy Cattle

**DOI:** 10.3390/ani16131998

**Published:** 2026-06-29

**Authors:** Pongsanun Khamta, Apirak Tadsorn, Aekaluck Leklerdsiriwong, Theerawat Swangchan-Uthai, Chaidate Inchaisri

**Affiliations:** 1 Center of Excellence in Data Innovation for Sustainable Livestock Production (CEDISLP), Department of Veterinary Medicine, Faculty of Veterinary Science, Chulalongkorn University, Bangkok 10330, Thailand; pongsanun.kha@gmail.com (P.K.); apirak.tx@gmail.com (A.T.); aekalek@gmail.com (A.L.); 2 Center of Excellence in Animal Fertility Chulalongkorn University (CU-AF), Department of Obstetrics, Gynaecology and Reproduction, Faculty of Veterinary Science, Chulalongkorn University, Bangkok 10330, Thailand; theerawat.s@chula.ac.th

**Keywords:** dairy cow, natural estrus, estrus detection, inertial measurement unit, gyroscope magnitude, dynamic body acceleration, posture-related behavior, standing estrus

## Abstract

Accurate estrus detection is essential for optimizing artificial insemination timing, but visual detection is limited by labor demands, intermittent observation, short estrus duration, and variable behavioral expression. Although inertial measurement unit (IMU) systems capture dynamic acceleration and rotational movement, phase-specific IMU-derived activity and posture-related changes during natural estrus remain insufficiently characterized. Therefore, this study evaluated these variables across an ovulation-anchored six-phase framework using video-derived behavioral observations and ultrasound-confirmed ovulation as biological reference standards. In this observational study, five dairy cows contributing eleven natural estrus cycles were monitored, yielding 285,337 time-aligned 10 s sensor observations that were summarized for phase-level analysis. Cow movement was recorded at 10 s intervals using neck-mounted tri-axial accelerometers and gyroscopes, while posture states, estrus-related behaviors, and ovulation timing were determined from continuous video recordings and 6 h transrectal ultrasonography. Extracted variables included signal vector magnitude, VeDBA, Gyro_mag, baseline-adjusted activity features, exploratory Combined Activity Index, posture proportions, and lying bout characteristics. VeDBA was highest during standing estrus, whereas Gyro_mag and the Combined Activity Index increased during pre-estrus and standing estrus. Standing estrus involved less lying, more standing and walking, and shorter lying bout duration. These findings identify candidate IMU-derived and posture-related variables for future standing-estrus differentiation models and potential insemination-timing support, pending validation in larger independent populations.

## Simple Summary

This study evaluated whether neck-mounted movement sensors could describe changes in activity and posture during natural estrus in dairy cows. Five cows with eleven natural estrus cycles were monitored using wearable sensors, continuous video observation, and ultrasound examination to confirm ovulation. Natural estrus was divided into six biological phases, allowing movement and posture-related behaviors to be compared before, during, and after standing estrus. Clear changes were observed during pre-estrus and standing estrus. During standing estrus, cows spent less time lying, more time standing and walking, and had shorter lying bouts. Body movement and head or neck rotation also increased during pre-estrus and standing estrus. These findings suggest that wearable movement sensors can help identify estrus-related behavioral changes during natural estrus. The identified activity and posture-related variables should be considered candidate features for future estrus-monitoring models and require validation in larger dairy cow populations before they are applied to support artificial insemination timing.

## 1. Introduction

Accurate estrus detection is a critical component of reproductive management in dairy herds because timely identification of cows in estrus supports appropriate timing of artificial insemination and improves reproductive efficiency [1,2]. Failure to detect estrus at the appropriate time can reduce conception rates, prolong calving intervals, and increase economic losses associated with reduced fertility performance [3,4]. Estrus in dairy cows is characterized by physiological and behavioral changes, including standing to be mounted, increased locomotor activity, mounting-related behavior, restlessness, and altered resting patterns [2,5]. Although visual observation remains widely used, its effectiveness is limited by labor requirements, observation frequency, housing conditions, short estrus duration, and variation in behavioral expression among cows [1,6]. Consequently, automated monitoring technologies have become increasingly important under practical farm conditions because they enable continuous detection of behavioral changes that may be missed by intermittent visual observation [6,7,8].

Precision dairy technologies, including pedometers, collars, ear tags, and wearable sensors, have been widely used to monitor activity and reproductive status in dairy cattle [2,9,10]. However, many estrus-monitoring studies have relied primarily on general activity measures or accelerometer-derived movement intensity to distinguish estrus from non-estrus periods, which can overlook coordinated changes in locomotion, mounting-related activity, social interaction, and resting behavior [11,12]. Inertial measurement unit (IMU) systems, which combine accelerometer and gyroscope sensors, provide more comprehensive behavioral information by capturing both linear acceleration and rotational movement [13]. Accelerometer-derived features mainly describe linear movement intensity, whereas gyroscope-derived features capture angular or rotational movement, particularly head and neck rotation when sensors are mounted on the collar [13,14,15]. This rotational component is biologically relevant during estrus because restlessness, orientation changes, sniffing, chin resting, mounting attempts, and social interactions can involve coordinated head and neck movements that are not fully represented by acceleration magnitude alone [2,5,16,17]. Previous IMU-based studies in cattle have demonstrated the usefulness of accelerometer- and gyroscope-derived variables for classifying basic behaviors such as lying, standing, walking, feeding, and ruminating [13,14,15]. In addition, sensor-based estrus studies have evaluated activity changes, behavioral patterns, or estrus detection using activity monitors, ear-mounted sensors, and automated monitoring systems [6,7,11,18,19,20,21]. However, most previous studies focused on broad estrus versus non-estrus comparisons, general activity increases, or basic behavior classification rather than evaluating how sensor-derived movement features change throughout the biological progression of natural estrus [6,11]. In particular, limited information is available on phase-specific changes in dynamic body acceleration, gyroscope-derived rotational movement, posture allocation, and lying-bout characteristics across ovulation-anchored phases of natural estrus.

This limitation is important because natural estrus is a dynamic process rather than a single uniform event. Behavioral expression changes progressively across the peri-estrus period, and the relationship between behavioral estrus and ovulation timing varies among cows [2,5,22]. Therefore, evaluating estrus only as “estrus” versus “non-estrus” may obscure biologically meaningful temporal patterns in activity and posture-related behavior, particularly across the transition from baseline behavior to pre-estrus, standing estrus, peri-ovulation, and post-ovulation recovery [1,19]. Accordingly, a phase-based approach can provide a more detailed understanding of how sensor-derived behavioral variables change throughout natural estrus. From a reproductive-management perspective, phase-based characterization can help identify whether sensor-derived changes occur before, during, or after standing estrus, which is important because the timing of standing estrus is biologically related to subsequent ovulation [5,20,22]. Therefore, identifying phase-specific activity and posture patterns can provide a foundation for future monitoring models designed to support estrus-alert interpretation and insemination-timing decisions [21,22].

Despite increasing interest in sensor-based estrus monitoring, limited evidence is available on how IMU-derived dynamic acceleration, rotational movement, posture allocation, and lying-bout characteristics change across ovulation-anchored phases of natural estrus. This gap is important because estrus is a dynamic biological process, and broad estrus versus non-estrus comparisons can obscure temporal changes occurring before, during, and after standing estrus.

To address this gap, the present study characterized IMU-derived activity features and posture-related behaviors across six biologically defined phases of natural estrus: normal, pre-estrus, standing estrus, late estrus, peri-ovulation, and early post-ovulation. These phases were defined using video-derived behavioral signs together with ultrasound-confirmed ovulation to provide a biologically meaningful temporal classification of estrus progression. Therefore, this exploratory descriptive study aimed to characterize phase-specific changes in IMU-derived activity features and posture-related behaviors during natural estrus in dairy cows and to identify candidate variables for future estrus-monitoring models, rather than to validate an estrus-detection algorithm.

## 2. Materials and Methods

This study was approved by the Institutional Animal Care and Use Committee (IACUC) of the Faculty of Veterinary Science, Chulalongkorn University, Thailand (Protocol No. 2031047), and was conducted in accordance with institutional regulations and the Ethical Principles and Guidelines for the Use of Animals for Scientific Purposes established by the National Research Council of Thailand.

### 2.1. Study Design and Animal Population

This observational study characterized inertial measurement unit (IMU)-derived activity features and posture-related behaviors across biologically defined phases of natural estrus in dairy cows under routine farm management conditions.

Data were collected from August to October 2021 in the loose barn of the Farm Animal Hospital, Faculty of Veterinary Science, Chulalongkorn University, Nakhon Pathom, Thailand. The monitored herd consisted of Holstein–Friesian crossbred dairy cows with approximately 75–87.5% Holstein–Friesian genetics. Cows were housed in a 30 × 15 m concrete-floored enclosure that allowed free movement and expression of natural behaviors. The housing facility and monitoring area are shown in Appendix C, Figure A4. Cows were fed a total mixed ration comprising 80% roughage and 20% concentrate on a dry matter basis at 2.5% of body weight, with feed provided twice daily at 09:00 and 14:00 h. Clean drinking water was available ad libitum.

Cows were selected from the monitored herd based on clinical health assessment, reproductive status evaluation, evidence of cyclic reproductive activity, and availability of complete synchronized IMU, video-based behavioral, and ultrasound-confirmed ovulation records. Eligible animals were non-production, non-pregnant dairy cows that expressed natural estrus during the monitoring period. The final study population included primiparous and multiparous cows, with parity ranging from 1 to 4. Because all included cows were non-production animals, milk yield, lactation stage, and days postpartum were not applicable as analytical variables. Cows or cycles were excluded if sensor, video, or ovulation-confirmation records were incomplete, or if health or reproductive abnormalities could affect natural estrus assessment.

After data screening, the final analytical dataset included five dairy cows contributing eleven natural estrus cycles. Although the dataset contained 285,337 time-aligned 10 s observations, the biological sample size was limited to five cows and eleven cow–cycle observations. Cow 1, Cow 2, and Cow 3 each contributed three cycles, whereas Cow 4 and Cow 5 each contributed one cycle. Each cow–cycle combination was considered the biological unit for phase assignment, and variables were summarized at the cow–cycle–phase level for statistical analysis.

During the monitoring period, neck-mounted IMU devices continuously recorded movement activity, while video observations and reproductive examinations provided behavioral and reproductive reference data. All study procedures were observational and did not interfere with routine animal handling, feeding, estrus observation, ovarian ultrasonography, or reproductive monitoring conducted at the facility.

### 2.2. Sensor Configuration and Placement

Cow activity was measured using a custom-built neck-mounted IMU device equipped with a tri-axial accelerometer and tri-axial gyroscope sensor (MPU-6050, InvenSense Inc., San Jose, CA, USA). The accelerometer quantified linear acceleration along three orthogonal axes, whereas the gyroscope captured angular velocity around the same axes, providing complementary information on translational and rotational motion.

The device continuously recorded raw acceleration and angular velocity signals along the X-, Y-, and Z-axes, which were stored with corresponding timestamps as mean values over consecutive 10 s intervals, equivalent to an effective sampling frequency of 0.1 Hz. This recording strategy was selected to support continuous long-duration monitoring across the full natural estrus period and to generate stable activity summaries suitable for phase-level characterization. Because the objective of the present study was to describe changes in activity and posture-related behavior across biologically defined estrus phases, rather than to detect individual short-duration estrus events, the 10 s aggregation interval was considered appropriate for the exploratory analytical framework. However, this sampling strategy reduces temporal resolution and can attenuate brief or rapid behavioral events, including individual mounting attempts, short standing-to-be-mounted events, abrupt head–neck movements, or transient social interactions; therefore, higher-frequency IMU data are generally more suitable for event-level behavior classification and detection [23,24]. Accordingly, the IMU-derived features in this study should be interpreted as indicators of broader phase-level movement patterns rather than event-level behavioral detections.

Each IMU device was enclosed in a protective housing and securely attached to the right side of the neck collar of each cow to continuously acquire head and neck movement data. The sensor axes were oriented relative to the cow’s body as follows: the X-axis represented the forward–backward direction, the Y-axis the vertical direction, and the Z-axis the lateral direction. The neck-collar location was selected because it is practical under farm conditions, compatible with collar-based monitoring systems, and suitable for capturing head, neck, and upper-body movements associated with posture changes, locomotion, restlessness, social interaction, and mounting-related activity [14,15,25,26]. Compared with leg-mounted sensors, neck-mounted sensors are generally less specific for direct detection of lying–standing transitions but provide broader information on upper-body and head–neck movement. Compared with ear-mounted sensors, neck-mounted sensors are less sensitive to small ear movements but can capture collar-level rotational and translational movement associated with whole-body activity [10,11,18,19]. Therefore, neck placement was considered appropriate for phase-level characterization of estrus-associated activity and posture-related behavioral changes in this study.

Timestamped accelerometer and gyroscope records enabled synchronization with video-based behavioral observations and reproductive reference data, including estrus signs, estrus scores, standing-to-be-mounted events, and ultrasound-confirmed ovulation timing.

### 2.3. Conceptual Framework of the Study

**Figure 1:**
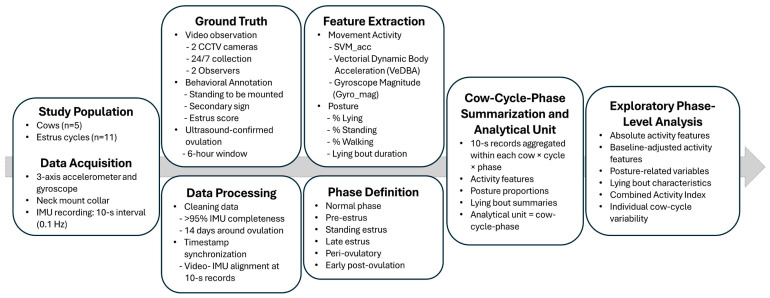
Study design and cow–cycle–phase analytical workflow for evaluating IMU-derived activity and posture-related behavioral changes across natural estrus phases in dairy cows. Continuous IMU records, video-derived behavioral observations, and ultrasonographic ovulation data were synchronized, assigned to biologically defined estrus phases, summarized at the cow–cycle–phase level, and used for exploratory phase-level comparisons.

The conceptual framework of this study was designed to integrate animal monitoring, reference data generation, sensor data preparation, feature extraction, estrus phase assignment, data summarization, and statistical analysis into a structured analytical workflow. This framework enabled IMU-derived movement features and posture-related behavioral variables to be interpreted in relation to biologically defined phases of natural estrus. The overall workflow is shown in Figure 1.

As illustrated in Figure 1, the workflow began with continuous sensor-based monitoring and video observation, followed by the preparation of synchronized sensor, behavioral, and reproductive reference datasets. The processed dataset was subsequently used for feature extraction, estrus phase classification, cow–cycle–phase-level summarization, and phase-level statistical analyses.

### 2.4. Data Acquisition and Ground-Truth Observation

#### 2.4.1. Video-Based Behavioral Observation

Reference behavioral data were obtained from continuous closed-circuit television (CCTV) footage. The loose-barn enclosure was monitored using two fixed CCTV cameras positioned at different elevated locations to provide complementary views of the housing and monitoring area, maximize visual coverage, and reduce blind spots. Video recording was conducted continuously throughout the monitoring period, including both daytime and nighttime observations. During nighttime and low-light periods, visibility was maintained using facility lighting and the low-light recording capability of the CCTV system. For each cow–cycle, video observation covered the full analytical period, beginning during the normal baseline phase before estrus-related behavioral changes were detected and continuing through the early post-ovulation phase. Daytime and nighttime CCTV views of the housing facility, together with a schematic layout showing camera positions, are provided in Appendix C, Figure A4.

Two trained observers reviewed the footage and classified cow behaviors according to a predefined ethogram. Before formal annotation, both observers underwent joint training and annotated a pilot dataset to harmonize behavioral definitions and interpretation criteria. Inter-observer reliability was assessed before consensus resolution using Cohen’s kappa on a randomly selected 5% subset of video segments sampled across cows and time periods to ensure behavioral and temporal diversity. The resulting Cohen’s kappa value was 0.84, indicating strong agreement between observers. Ambiguous behaviors or discrepancies were subsequently rechecked and resolved through discussion and consensus. Annotation was conducted blind to the study hypotheses to reduce observer bias. To improve transparency, the observer recording template used for basal behavior and estrus-related behavior annotation is provided in Appendix D, Figure A5.

#### 2.4.2. Behavioral Annotation and Estrus Scoring

The main behavioral categories analyzed were lying, standing, and walking, which represented biologically interpretable posture and activity states relevant to estrus-associated changes. Although several behaviors were initially annotated, only lying, standing, and walking were retained as primary posture-related outcomes for the present phase-level analysis. Eating and drinking were excluded from the primary outcome set because the number of observed instances was insufficient for reliable phase-level comparison, whereas ruminating was excluded because it overlapped with other posture states and involved ambiguous neck movements that reduced annotation reliability.

In the present study, the IMU system was not used to directly classify individual estrus-related behaviors. Continuous video observation served as the reference standard for posture states and estrus-related signs, including standing-to-be-mounted behavior and secondary estrus indicators. IMU records were used to extract acceleration- and gyroscope-derived activity features and to evaluate their phase-specific changes across natural estrus. Therefore, estrus-phase assignment was based on video-derived behavioral evidence and ultrasound-confirmed ovulation rather than automated IMU classification of estrus behaviors.

Estrus-related behavioral signs were identified from continuous video records using a scoring system adapted from previously published visual estrus scoring frameworks [17,27]. This scoring system was not newly developed for the present study but was adapted to support consistent classification of standing estrus and secondary estrus signs from continuous video observations. Standing-to-be-mounted behavior was assigned the highest score of 100 points and was used as the principal indicator of standing estrus. Secondary estrus indicators were scored according to their behavioral relevance, including mounting the head side of another cow (45 points), mounting or attempting to mount another cow (35 points), chin resting (15 points), sniffing the vulva or vaginal region of another cow (10 points), being mounted without standing (10 points), restlessness (5 points), and flehmen response (3 points). These scores were used to distinguish standing estrus from secondary estrus-related behavioral expression during phase classification.

#### 2.4.3. Estrus Event Alignment and Ovulation Confirmation

Estrus-related events were identified from continuous video records and aligned with the corresponding 10 s IMU records using timestamp synchronization. Standing estrus was classified when standing-to-be-mounted behavior was observed and assigned an estrus score ≥100. Secondary indicators were recorded when one or more secondary estrus signs were observed and the corresponding estrus score was ≥15 and <100. To reduce misclassification of isolated or ambiguous events, a secondary indicator was considered valid for pre-estrus identification only when another secondary indicator occurred within the following 2 h. Estrus scores were assigned according to the presence and temporal clustering of these indicators, following the principle that estrus expression should be assessed using both standing behavior and secondary signs rather than activity level alone.

Ovulation was determined by transrectal ovarian ultrasonography. Ultrasonographic monitoring was initiated when standing-to-be-mounted behavior was first detected during visual observation conducted at 6 h intervals. After the first standing-to-be-mounted event was detected, ovarian examinations were performed every 6 h, at approximately 05:00, 11:00, 17:00, and 23:00 h, to monitor the dominant follicle until it was no longer visible. Thus, the reproductive monitoring period for each cow–cycle covered the interval from the first detected standing-to-be-mounted behavior to the first ultrasound examination at which disappearance of the dominant follicle confirmed ovulation. The duration of ultrasonographic monitoring varied among cow–cycle combinations, ranging from 16.60 to 59.46 h after the first detected standing-to-be-mounted behavior.

For each cycle, ovulation was estimated within a 6 h interval defined by the last ultrasound examination at which the dominant follicle was visible and the first subsequent examination at which it was no longer detected. Because ovulation could not be determined as an exact time point, it was operationally assigned as the ovulation midpoint, calculated as the midpoint of the 6 h peri-ovulation window described in Table 1. This definition enabled temporal alignment of behavioral and IMU records while acknowledging the resolution limit of 6 h ultrasonographic monitoring [5,22].

### 2.5. Data Preprocessing and Cleaning

Raw IMU data were processed using Python (version 3.10; Python Software Foundation, Wilmington, DE, USA) [28] in Jupyter Notebook (version 7.0.8) through the Anaconda distribution. Data preparation included data importation, timestamp formatting, chronological sorting, missing-value inspection, removal of malformed entries, and assessment of time gaps.

Missing, invalid, or non-numeric sensor records were screened before feature extraction. Records with missing or invalid timestamps, missing sensor values, or non-numeric entries were excluded. Extreme sensor values were handled using IQR-based clipping with a conservative 5 × IQR threshold; values outside Q1 − 5 × IQR to Q3 + 5 × IQR were clipped to the corresponding boundary rather than removed. This conservative threshold was selected to reduce transient sensor artifacts while preserving biologically meaningful high-activity values associated with estrus-related movement. Overall, 105 records were excluded, 12,780 sensor values were clipped, and 285,337 time-aligned 10 s observations were retained for phase-level analysis.

Data completeness was evaluated at the cow–cycle level and calculated as the proportion of observed IMU records relative to the expected number of records within each analytical window. The expected number of records was estimated from the duration of the analytical window and the expected 10 s recording interval. Only cycles with sufficient IMU coverage were retained, with the target completeness threshold defined as >95% of expected IMU records. Time gaps were identified from the time difference between consecutive records. Given the expected 10 s recording interval, gaps exceeding 120 s were classified as extended gaps. Extended gaps were assessed to minimize potential bias in duration-based posture variables, including behavioral duration, posture percentage, and lying bout characteristics.

For each cow–cycle combination, the analytical window was defined relative to ovulation and extended from the normal pre-estrus baseline to the early post-ovulation period. Following cleaning, observations were assigned to estrus phases according to the rule-based definitions described in Section 2.6.

### 2.6. Estrus Phase Definition

Each natural estrus cycle was divided into six biologically defined phases using video-derived behavioral signs, estrus scores, and ultrasound-confirmed ovulation, as described in Section 2.4. The study-specific six-phase classification was designed to represent the temporal progression of natural estrus from baseline behavior to secondary estrus signs, standing estrus, the post-standing period, ovulation, and early post-ovulation recovery. This framework was supported by previous studies showing that estrus behavior changes sequentially before, during, and after standing estrus, with standing-to-be-mounted behavior serving as the primary behavioral sign of estrus [5,17,27,29]. In addition, ovulation generally occurs after the onset and end of standing estrus, although the interval varies among cows and studies [5,20,22,30]. Therefore, this phase-based structure enabled sensor-derived activity and posture-related behavioral changes to be interpreted according to their temporal relationship with standing estrus and ovulation.

For each cow–cycle, phase boundaries were assigned using a rule-based approach integrating video-derived estrus signs and ultrasound-confirmed ovulation. The normal phase represented the baseline period before valid estrus-associated behavioral signs. Pre-estrus began at the first valid secondary estrus sign, defined as an estrus score ≥ 15 and <100 followed by another secondary sign within 2 h. Standing estrus extended from the first to the last standing-to-be-mounted event or observation window with an estrus score ≥ 100. Late estrus began after the cessation of standing estrus and continued until the start of the ultrasound-confirmed peri-ovulation window. Peri-ovulation was defined as the 6 h interval between the last observation of the dominant follicle and the first ultrasound examination in which it was no longer visible. Early post-ovulation began immediately after the peri-ovulation window. Because these boundaries were assigned independently for each cow–cycle according to behavioral expression and ovulation timing, phase durations differed among cycles. The operational definition of each phase is provided in Table 1.

These definitions were applied independently to each cow–cycle combination. Phase duration was calculated in hours, and sensor-derived activity features and posture-related variables were summarized at the cow–cycle–phase level. This structure enabled phase-level evaluation of movement and behavioral changes while preserving the cow–cycle organization of the dataset.

### 2.7. Feature Extraction

IMU-derived activity features and posture-related variables were extracted to characterize movement and behavioral changes across biologically defined estrus phases. The feature set included absolute movement measures, baseline-adjusted activity features, an exploratory Combined Activity Index, and posture-based outcomes. The activity and postural variables are summarized in Table 2.

Absolute activity measures were calculated from accelerometer and gyroscope signals and included signal vector magnitude of acceleration, vectorial dynamic body acceleration, and gyroscope magnitude. These measures described linear movement intensity, dynamic body acceleration, and rotational activity [14,15,31,32,33]. Posture-based outcomes were derived from annotated behavioral labels and included the proportions of time spent lying, standing, and walking, together with lying bout characteristics, which are commonly used to describe resting and activity patterns in dairy cows [34,35,36,37].

#### 2.7.1. Signal Vector Magnitude of Acceleration

Signal vector magnitude of acceleration (SVM_acc) was calculated from tri-axial accelerometer signals to quantify overall linear movement intensity. For each timestamp, acceleration values from the X, Y, and Z axes were combined using the Euclidean norm:

$$SVM\_acc=\sqrt{\left({AccX}^{2}{+AccY}^{2}{+AccZ}^{2}\right)}$$

where AccX, AccY, and AccZ represent acceleration values along the three orthogonal axes. Higher SVM_acc values indicate greater overall linear movement intensity.

#### 2.7.2. Vectorial Dynamic Body Acceleration

Vectorial dynamic body acceleration (VeDBA) was calculated to quantify dynamic movement after reducing the influence of static acceleration. For each accelerometer axis, static acceleration was estimated using a rolling mean and subtracted from the raw acceleration signal to obtain the dynamic acceleration component. This procedure was used to reduce the influence of gravity and sensor orientation, allowing VeDBA to better represent movement-related acceleration [31,32,33]. VeDBA was then calculated as follows:

$$VeDBA=\sqrt{\left({DBAx}^{2}{+DBAy}^{2}{+DBAz}^{2}\right)}$$

where DBAx, DBAy, and DBAz represent dynamic body acceleration along each axis. VeDBA was used as an activity-related feature because it reflects dynamic body movement and may increase during active estrus-related behaviors.

#### 2.7.3. Gyroscope Magnitude

Gyroscope magnitude (Gyro_mag) was calculated from tri-axial gyroscope signals to quantify rotational movement intensity. For each timestamp, angular velocity values from the X, Y, and Z axes were combined using the Euclidean norm:

$$Gyro\_mag=\sqrt{\left({GyroX}^{2}{+GyroY}^{2}{+GyroZ}^{2}\right)}$$

where GyroX, GyroY, and GyroZ represent angular velocity around the three axes. Gyro_mag was included because rotational movement of the head and neck may increase during restlessness, walking, mounting attempts, or other estrus-related activities.

#### 2.7.4. Baseline-Adjusted Activity Features

Baseline-adjusted activity features were calculated to account for individual differences in normal activity among cows. The normal phase was used as the cow-specific reference period because estrus-related activity is commonly interpreted as a deviation from each cow’s baseline activity level rather than as an absolute activity threshold alone [1,7,8]. For each cow–cycle, the mean and standard deviation of each activity feature during the normal phase were calculated and used to generate baseline-standardized and baseline-difference transformations.

Baseline-standardized features were calculated as follows:

$${Base}_{z}=\frac{X_{t}-\mu_{baseline}}{\sigma_{baseline}}$$

where Xt represents the activity value at time t, μ baseline represents the cow-specific baseline mean during the normal baseline phase, and σ baseline represents the cow-specific baseline standard deviation during the normal baseline phase. These values indicate how many standard deviations each observation deviated from the cow’s normal baseline activity level. Baseline-difference features were calculated as follows:

$$Base_{diff}=X_{t}-\mu_{baseline}$$

This transformation quantified the absolute deviation from baseline while preserving the original feature scale. Baseline-adjusted features were generated for SVM_acc, VeDBA, and Gyro_mag.

#### 2.7.5. Exploratory Combined Activity Index

An exploratory Combined Activity Index was developed to integrate dynamic body acceleration and rotational movement into a single composite measure. VeDBA and Gyro_mag were selected because they represent complementary movement dimensions: VeDBA reflects dynamic body acceleration, whereas Gyro_mag reflects rotational head–neck movement. Because estrus expression is associated with coordinated changes in locomotor activity, restlessness, social interaction, and mounting-related behavior, combining these two variables was expected to provide a broader representation of estrus-associated movement than either variable alone [2,5,11,25,26].

Before combination, VeDBA and Gyro_mag were normalized using min–max normalization to place both variables on a comparable 0–100 scale. Normalization was necessary because VeDBA and Gyro_mag were measured on different numerical scales and units; therefore, min–max normalization allowed both variables to contribute to the weighted composite on a comparable scale. Normalization was calculated as follows:

$$X_{norm}=\frac{X-X_{min}}{X_{max}-X_{min}}\times 100$$

where X represents the observed value, Xmin represents the minimum value, and Xmax represents the maximum value of the feature.

The Combined Activity Index was calculated as a weighted composite of normalized VeDBA and normalized Gyro_mag:

$$Combined\;Activity\;Index=\left(\omega_{1}\times {VeDBA}_{norm}\right)+(\omega_{2}\times {Gyro}_{norm})$$

where $\omega_{1}+\omega_{2}=1$.

Several weighting combinations were explored, including VeDBA80:Gyro20, VeDBA70:Gyro30, VeDBA60:Gyro40, VeDBA50:Gyro50, VeDBA40:Gyro60, VeDBA30:Gyro70, and VeDBA20:Gyro80. The VeDBA40:Gyro60 weighting was selected for descriptive presentation because it provided a biologically meaningful balance between dynamic body acceleration and rotational movement. Standing estrus involves both increased locomotor activity and rotational head–neck movements associated with restlessness, social interaction, body orientation changes, and mounting-related activity. Therefore, retaining a meaningful contribution from VeDBA preserved acceleration-derived information, whereas the slightly greater contribution from Gyro_mag reflected the clearer phase-related response of rotational movement in the present dataset. This balance reduced dependence on a single sensor modality and provided a clear, biologically interpretable phase-related pattern. Higher values indicated greater combined dynamic and rotational movement. However, this weighting was selected descriptively within the present dataset and was not statistically optimized or externally validated. Therefore, the Combined Activity Index should be interpreted as an exploratory composite indicator rather than as a finalized estrus-detection score or practical decision threshold. The final Combined Activity Index was calculated as follows:

Combined Activity Index = 0.40 × normalized VeDBA + 0.60 × normalized Gyro_mag

Higher values indicated greater combined dynamic and rotational movement. Because the Combined Activity Index was developed and selected within the present dataset, it should be interpreted as an exploratory composite indicator of estrus-related activity intensity rather than an optimized or externally validated index. Validation in larger independent populations is required before general application.

#### 2.7.6. Posture Duration

Posture-related behavioral features were derived from video-based behavioral annotations. The primary behavioral categories used to generate posture-related variables were lying, standing, and walking. Although eating was defined in the ethogram, it was not included as a primary posture variable in the phase-level analysis because the main focus of the study was on resting, standing, and locomotor activity during estrus. Behavioral definitions are summarized in Table 3.

The primary posture-related variables were the percentages of time spent lying, standing, and walking within each estrus phase. Proportional variables were used instead of raw duration totals because phase durations differed among cow–cycle combinations. For each cow–cycle–phase unit, the percentage of each behavior was calculated as follows:

$$Percent\;behavior=\frac{Duration\;of\;behavior\;within\;phase}{Total\;phase\;duration}\times 100$$

Lying bout characteristics were also calculated to describe resting patterns across estrus phases. A lying bout was defined as a continuous period during which a cow was classified as lying. Lying bout rate was expressed as the number of lying bouts per hour, whereas mean lying bout duration was expressed in minutes per bout. These variables were included to characterize changes in the frequency and duration of resting episodes, rather than total lying time alone.

### 2.8. Data Summarization and Analytical Unit

To minimize pseudoreplication, the dataset was aggregated at the cow–cycle–phase level prior to inferential testing. Each cow–cycle–phase unit served as the analytical unit rather than treating individual timestamp-level measurements as independent observations. The workflow for converting synchronized 10 s records into cow–cycle–phase analytical summaries is illustrated in Figure 1.

Activity and posture-related variables were summarized within each cow–cycle–phase unit. Activity summaries included mean SVM_acc, mean VeDBA, mean Gyro_mag, baseline-standardized activity features, and baseline-difference activity features. Posture-related summaries included the percentages of time spent lying, standing, and walking, lying bout rate per hour, and mean lying bout duration expressed in minutes.

The resulting phase-level dataset served as the basis for inferential comparisons among estrus phases. This approach allowed comparisons to reflect biological variation among cows and estrus cycles rather than differences in raw sensor-record counts. It also reduced the risk of artificially inflated sample sizes from repeated within-cow time-series measurements.

### 2.9. Statistical Analysis

Statistical analyses were conducted in RStudio (Build 2023.06.1+524; Posit Software, PBC, Boston, MA, USA) [38] using the cow–cycle–phase summary described in Section 2.8.

Statistical analyses were conducted as exploratory phase-level comparisons using cow–cycle–phase summaries as the analytical unit. Timestamp-level sensor records were aggregated before statistical testing to reduce pseudoreplication.

The cow–cycle–phase summary was selected as the analytical unit to reduce pseudoreplication from repeated 10 s sensor observations. Because the final dataset included eleven natural estrus cycles from five cows, the number of higher-level biological units was limited for estimating random-effect variance components in linear mixed-effects models. Therefore, the statistical analysis was interpreted as exploratory phase-level comparison rather than definitive population-level inference. Future studies with larger numbers of cows and estrus cycles are required to support more robust mixed-effects modelling of cow- and cycle-level random effects.

Because the effective biological sample consisted of 11 estrous cycles from 5 cows, mixed-effects models were not applied due to limited higher-level replication for stable random-effect estimation. Therefore, statistical comparisons were interpreted as descriptive and hypothesis-generating rather than confirmatory population-level inference. Descriptive statistics were calculated for each IMU-derived activity feature and posture-related measure across estrus phases.

Before inferential testing, each phase-level measure was assessed for normality using the Shapiro–Wilk test. When the normality assumption was met, phase-related variation was evaluated using one-way analysis of variance (ANOVA), followed by Tukey’s honestly significant difference post hoc test. When the normality assumption was not met, the Kruskal–Wallis test was applied, followed by Bonferroni-adjusted pairwise Wilcoxon rank sum tests.

Statistical significance was defined as *p* < 0.05. Compact letter displays were generated for figures and summary tables to indicate significant phase differences. Phases sharing a letter were considered not significantly different, whereas phases with different letters were considered significantly different. Accordingly, *p*-values were used to support descriptive phase-level interpretation rather than definitive population-level inference.

Graphical outputs included phase-level boxplots with compact letter displays, trajectories of activity and posture-related variables relative to ovulation, and supplementary plots for individual cow–cycle combinations. These visualizations were used to evaluate overall phase-level patterns and individual variability in estrus-associated behavioral dynamics.

## 3. Results

### 3.1. Data Structure and Phase Distribution

A total of 285,337 time-aligned 10 s observations across 11 natural estrus cycles from 5 dairy cows were retained after preprocessing (Table 4). These timestamp-level observations were summarized at the cow–cycle–phase level before statistical analysis. Because all 11 estrus cycles contributed data to each of the six biologically defined phases, each phase-level comparison was based on 11 cow–cycle–phase summary units, resulting in 66 cow–cycle–phase summaries overall. The timestamp-level observations were distributed unevenly across phases because phase duration differed among cow–cycle combinations. The normal phase (Phase 1) represented the largest proportion of the dataset, with 88,100 observations (30.9%). This was followed by the early post-ovulation phase (Phase 6; 22.4%) and the late estrus phase (Phase 4; 19.4%). The pre-estrus phase (Phase 2; 8.4%) and peri-ovulation phase (Phase 5; 6.4%) accounted for the smallest proportions of the dataset. The number of timestamp-level observations also varied among cows and estrus cycles, ranging from 7249 in Cycle 7 (Cow 3) to 32,534 in Cycle 11 (Cow 5). The unequal number of estrus cycles among cows reflected differences in the availability of complete eligible natural estrus cycles during the monitoring period. Cows 1–3 contributed three eligible cycles each, whereas Cows 4 and 5 contributed one eligible complete cycle; subsequent health-related issues during the experiment prevented additional eligible cycles from being collected from these cows. Therefore, only the complete eligible cycles with synchronized IMU, video, and ultrasound-confirmed ovulation records were included in the final analysis.

### 3.2. Estrus Phase Duration Characteristics

Estrus phase durations are summarized in Table A1. The normal phase (Phase 1) had the longest mean duration (26.92 ± 7.41 h), whereas the peri-ovulation phase (Phase 5) had the shortest mean duration (5.92 ± 0.24 h). Duration ranges varied across estrus phases. The pre-estrus phase (Phase 2; 8.74 ± 6.69 h) and late estrus phase (Phase 4; 17.77 ± 8.56 h) exhibited greater variation among estrus cycles. Standing estrus (Phase 3) had a mean duration of 11.41 ± 5.85 h.

### 3.3. Changes in Absolute Activity Features Across Estrus Phases

**Figure 2:**
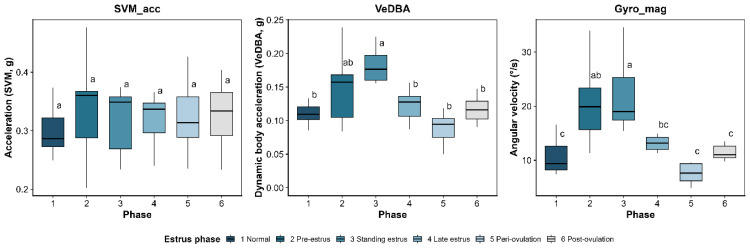
Absolute activity features across natural estrus phases. Footnote: Values are presented as mean ± SD. Different superscript letters indicate statistically significant differences between phases (*p* < 0.05).

Absolute activity features are presented in Figure 2 and Table A2. VeDBA and Gyro_mag showed significant differences across estrus phases, whereas SVM_acc showed no significant differences among phases. VeDBA was highest during standing estrus (Phase 3; 0.17 ± 0.05). Lower values were observed during the remaining phases. Elevated Gyro_mag values were observed during the pre-estrus phase (Phase 2; 20.19 ± 6.56) and standing estrus (Phase 3; 22.10 ± 6.85), whereas lower values occurred during the remaining phases. Overall, VeDBA and Gyro_mag increased from the normal phase to standing estrus and decreased during the late estrus, peri-ovulation, and post-ovulation phases.

Although significant phase differences were observed for VeDBA and Gyro_mag, these results should be interpreted as exploratory. Biological relevance was considered based on the magnitude and consistency of phase-related patterns rather than statistical significance alone. The clearer elevation of VeDBA and Gyro_mag during pre-estrus and standing estrus suggests that these features were more informative than SVM_acc for describing estrus-associated movement changes in the present dataset.

### 3.4. Baseline-Standardized and Baseline-Difference Activity Changes

**Figure 3:**
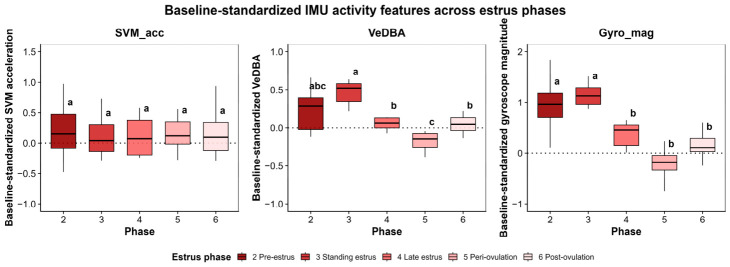
Baseline-standardized activity features across natural estrus phases. Footnote: Baseline-standardized values were calculated using cow-specific reference values from the normal phase. Superscript letters indicate significant differences between phases (*p* < 0.05).

**Figure 4:**
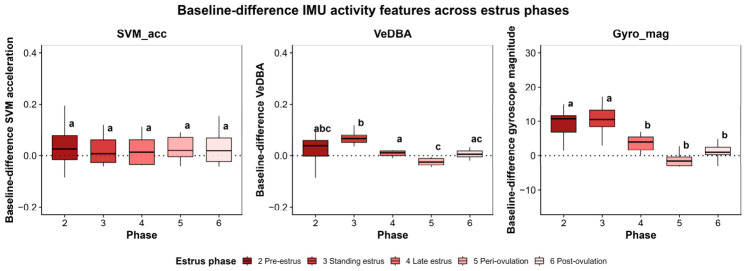
Baseline-difference activity features across natural estrus phases. Footnote: Baseline-difference values were calculated using cow-specific reference values from the normal phase. Superscript letters indicate significant differences between phases (*p* < 0.05).

Baseline-standardized and baseline-difference activity features are shown in Figure 3 and Figure 4, and Table A2. Baseline-standardized VeDBA and Gyro_mag showed significant differences across estrus phases, whereas baseline-standardized SVM_acc showed no significant differences among phases. Baseline-standardized SVM_acc distributions overlapped substantially among phases. The highest baseline-standardized VeDBA values were observed during standing estrus (Phase 3). Elevated baseline-standardized Gyro_mag values were observed during pre-estrus (Phase 2) and standing estrus (Phase 3). Lower values for both features were observed during the late estrus, peri-ovulation, and post-ovulation phases, including negative values during peri-ovulation. Baseline-difference VeDBA and Gyro_mag showed similar phase-related patterns, with elevated values during pre-estrus and standing estrus and lower values during peri-ovulation and post-ovulation phases. Baseline-difference SVM_acc showed no significant differences among phases. Overall, both baseline-standardized and baseline-difference activity features increased during pre-estrus and standing estrus and decreased during peri-ovulation and post-ovulation phases. These baseline-adjusted patterns were interpreted as exploratory indicators of phase-related deviation from each cow’s normal activity level rather than validated detection thresholds.

### 3.5. Exploratory Combined Activity Index Across Estrus Phases

**Figure 5:**
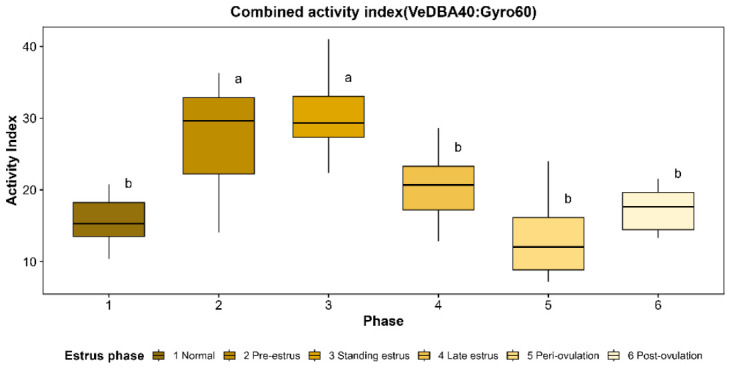
Exploratory Combined Activity Index across natural estrus phases using VeDBA40:Gyro60 weighting. Footnote: Values are presented as mean ± SD. Different superscript letters indicate statistically significant differences between phases (*p* < 0.05).

The exploratory Combined Activity Index (VeDBA40:Gyro60) is shown in Figure 5 and Table A2. The Combined Activity Index showed significant differences across estrus phases. Elevated Combined Activity Index values were observed during pre-estrus (Phase 2; 27.41 ± 7.34) and standing estrus (Phase 3; 30.36 ± 5.33). Lower values were observed during the normal, late estrus, peri-ovulation, and post-ovulation phases. Overall, the Combined Activity Index increased from the normal phase to standing estrus and decreased during the late estrus, peri-ovulation, and post-ovulation phases.

Alternative VeDBA and Gyro_mag weightings produced broadly similar phase-related patterns, with higher values during pre-estrus and standing estrus than during the remaining phases. The VeDBA40:Gyro60 weighting was retained for presentation because it provided a biologically interpretable balance between dynamic body acceleration and rotational movement. However, this weighting remains exploratory and requires validation in larger independent datasets.

### 3.6. Changes in Posture-Related Behavior Across Estrus Phases

**Figure 6:**
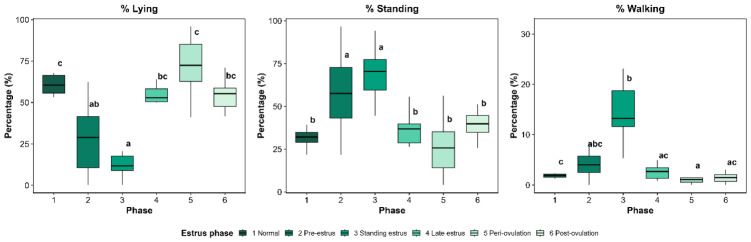
Posture-related behavior proportions across natural estrus phases. Footnote: Values represent percentage of time spent in each posture. Superscript letters denote significant differences between phases (*p* < 0.05).

Posture-related behavior proportions are presented in Figure 6 and Table A2. Lying, standing, and walking proportions showed significant differences across estrus phases. Lying proportion was lowest during standing estrus (Phase 3; 13.89 ± 9.71%) and highest during the peri-ovulation phase (Phase 5; 68.31 ± 24.94%). Standing proportion was highest during standing estrus (Phase 3; 70.16 ± 14.74%), whereas walking proportion also reached its highest value during standing estrus (13.70 ± 6.95%). Lower walking proportions were observed during the remaining phases. Overall, lying behavior decreased from the normal phase to standing estrus, whereas standing and walking behaviors increased. During the peri-ovulation and post-ovulation phases, lying behavior increased while standing and walking decreased.

### 3.7. Lying Bout Characteristics

**Figure 7:**
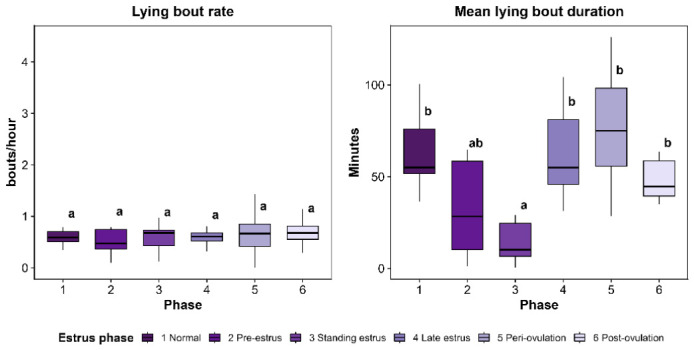
Lying bout characteristics across natural estrus phases. Footnote: Bout rate is expressed as bouts per hour. Bout duration is expressed in minutes per bout. Superscript letters indicate significant differences (*p* < 0.05).

Lying bout characteristics are presented in Figure 7 and Table A2. Lying bout rate did not differ significantly across estrus phases, whereas mean lying bout duration showed significant differences among phases. The shortest mean lying bout duration was observed during standing estrus (Phase 3; 22.33 ± 29.77 min). Higher values were observed during the normal, late estrus, peri-ovulation, and post-ovulation phases. Overall, mean lying bout duration decreased from the normal phase to standing estrus and increased during the late estrus, peri-ovulation, and post-ovulation phases.

### 3.8. Individual Cycle Variability in Estrus-Related Behavior

**Figure A1:**
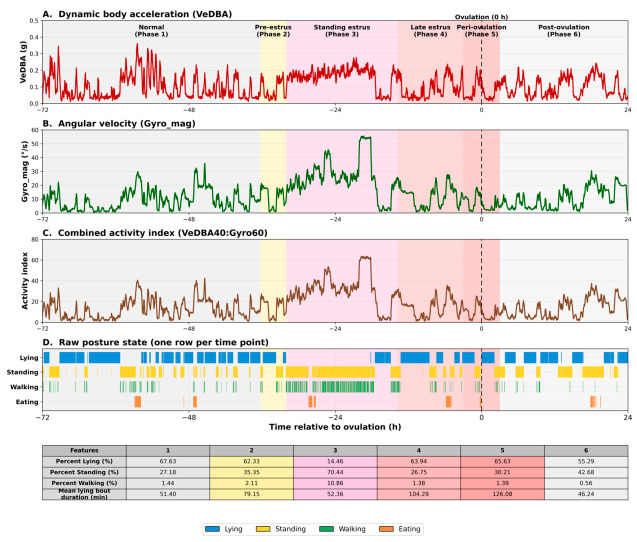
Time-series patterns of VeDBA, Gyro_mag, Combined activity index, posture states, and posture-related behavioral features across estrus phases in Cycle No.5 (Cow2).

**Figure A2:**
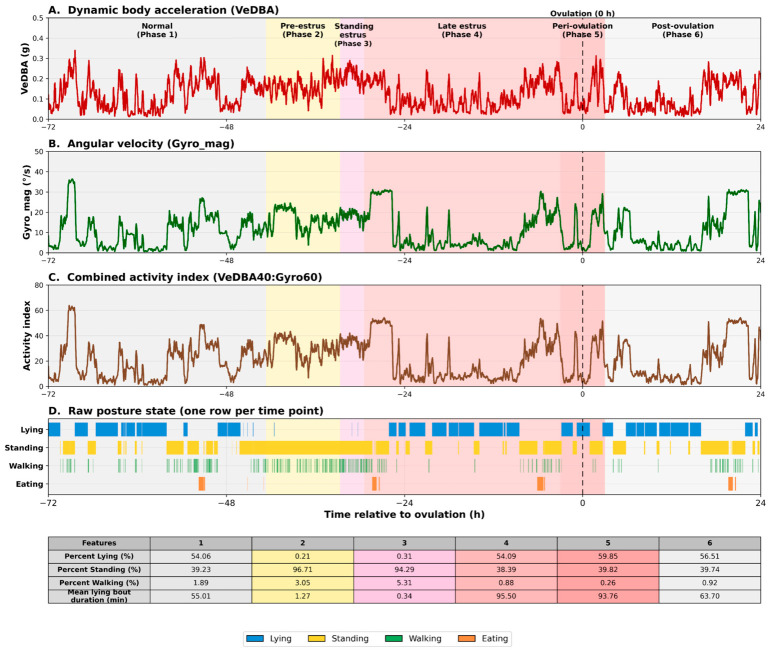
Time-series patterns of VeDBA, Gyro_mag, Combined activity index, posture states, and posture-related behavioral features across estrus phases in Cycle No.10 (Cow4).

**Figure A3:**
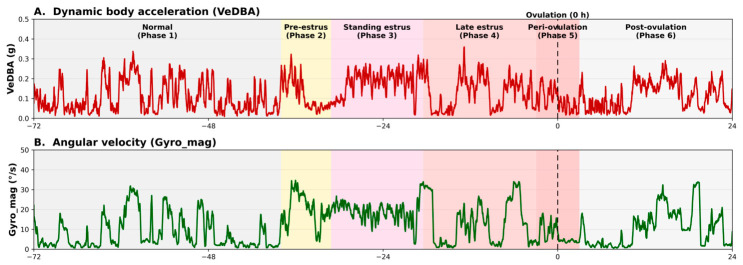

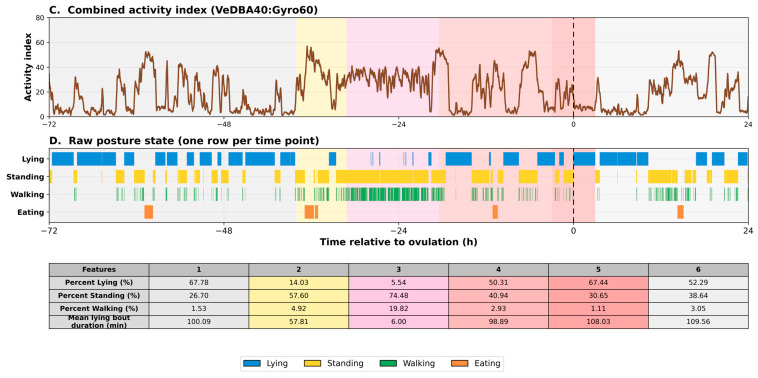
Time-series patterns of VeDBA, Gyro_mag, Combined activity index, posture states, and posture-related behavioral features across estrus phases in Cycle No.11 (Cow5).

**Figure A4:**
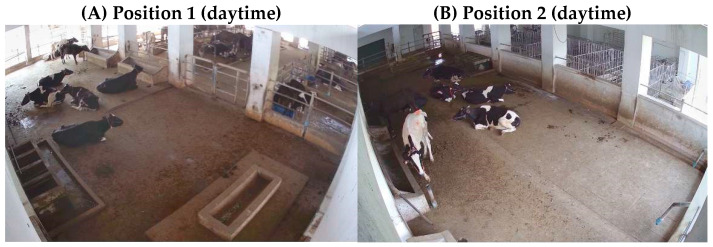

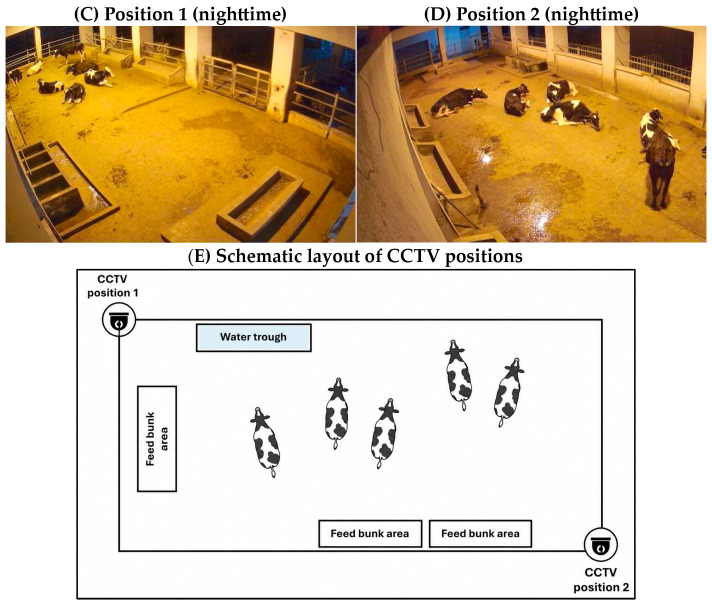
Daytime and nighttime CCTV views of the loose-barn housing facility and schematic positions of the CCTV cameras used for behavioral monitoring. Panels (**A**) and (**B**) show daytime CCTV views of the loose-barn enclosure from CCTV positions 1 and 2, respectively. Panels (**C**) and (**D**) show nighttime CCTV views from the same two camera positions. Panel (**E**) presents a schematic layout of the housing facility, including the positions of the two CCTV cameras, the water trough, and feed bunk areas. Together, these images illustrate the experimental environment and video-monitoring setup used for continuous behavioral observation during neck-mounted IMU monitoring and video-based behavioral annotation.

**Figure A5:**
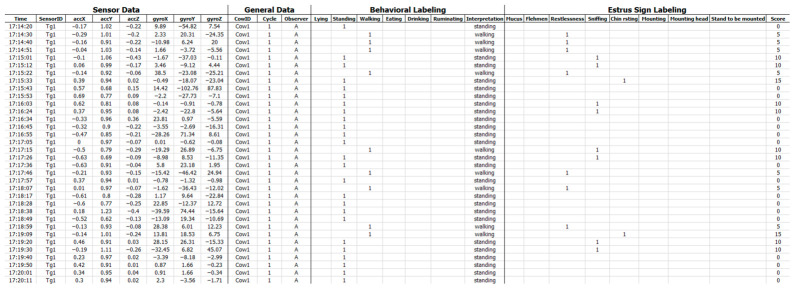
Example of the observer recording template used for basal behavior and estrus-related behavior annotation. Footnote: The template was used to record cow identification, observation time, and basal posture or activity states, including lying, standing, walking, and eating. The template was also used to record cow identification, observation time, estrus-related behavioral signs, and assigned estrus scores according to the predefined scoring system.

Individual time-series patterns of activity and posture-related behaviors are presented in Figure A1, Figure A2 and Figure A3. Considerable cow–cycle variability was observed in the magnitude and temporal expression of estrus-related activity and posture changes. Despite this variability, several consistent patterns were evident across individual cycles. VeDBA and Gyro_mag generally increased during pre-estrus and standing estrus, whereas lying proportion and mean lying bout duration tended to decrease during standing estrus and increase again during peri-ovulation or early post-ovulation. However, the magnitude and timing of these changes differed among cows and cycles. For example, Cycle No. 5 (Cow 2) showed pronounced increases in VeDBA and Gyro_mag together with high standing proportions during standing estrus. Cycle No. 10 (Cow 4) exhibited high standing proportions during both pre-estrus and standing estrus, whereas Cycle No. 11 (Cow 5) showed the highest walking proportion during standing estrus. These individual patterns indicate that estrus-related behavioral expression varied among cow–cycles, supporting the need for cow-specific baseline adjustment and cautious interpretation of pooled phase-level summaries.

## 4. Discussion

This exploratory study indicated that IMU-derived activity features and posture-related behaviors varied across biologically defined phases of natural estrus in dairy cows. The findings suggest that behavioral expression during natural estrus is dynamic rather than uniform across the peri-estrus period. The clearest behavioral differentiation was observed during pre-estrus and standing estrus, indicating that these phases are particularly relevant for future sensor-based estrus-monitoring model development. By representing estrus as a sequence of biologically defined phases rather than as a simple estrus versus non-estrus condition, the present study provided descriptive phase-specific information on movement intensity, rotational activity, posture allocation, and resting behavior before, during, and after standing estrus.

### 4.1. Phase-Specific IMU-Derived Activity Changes

Among the evaluated activity features, VeDBA and Gyro_mag showed more biologically informative responses than SVM_acc for characterizing estrus-related movement changes under the conditions of this study. VeDBA showed the greatest response during standing estrus, whereas Gyro_mag increased during both pre-estrus and standing estrus, indicating that dynamic acceleration and rotational movement captured different aspects of estrus expression. This pattern is consistent with previous studies reporting increased activity, restlessness, mounting-related behavior, and social interaction during estrus [1,2,5,11]. The phase-related patterns of VeDBA and Gyro_mag reflected their association with distinct movement components: VeDBA represents dynamic body acceleration after reducing the influence of static acceleration, whereas Gyro_mag captures rotational head and neck movements associated with restlessness, locomotion, and mounting-related interactions [15,31,32]. In contrast, SVM_acc includes both static and dynamic acceleration components and can be affected by posture, gravity-related acceleration, and neck position in a neck-mounted sensor configuration [24,33]. These findings indicate that dynamic acceleration and rotational movement features provide more specific information on estrus-associated movement changes than simple acceleration magnitude alone [23,39].

Sensor placement is an important consideration when interpreting these activity features. Because the IMU was mounted on the neck, Gyro_mag primarily reflected rotational head and neck movement, which can increase during restlessness, social investigation, locomotion, mounting attempts, and other estrus-related interactions. In contrast, leg-mounted sensors more directly capture lying, standing, stepping, and limb-associated locomotion, whereas ear-mounted sensors reflect ear and head movement and can also provide information related to activity and rumination [10,19,24,25]. Therefore, estrus-associated sensor features are partly placement-specific, and findings from neck-mounted IMU systems should not be assumed to be directly interchangeable with those from ear-tag, leg-mounted, or pedometer-based systems [8,9,19,25].

Baseline-adjusted activity features, including baseline-standardized and baseline-difference variables, improved the interpretation of estrus-related changes by expressing activity deviations relative to each cow’s normal activity level. This approach is consistent with previous automated estrus-monitoring studies, in which estrus detection was commonly based on identifying deviations from an individual cow’s usual activity pattern rather than relying only on fixed population-level thresholds [1,7,8]. This individual-reference approach is biologically relevant because activity expression during estrus varies among cows and is influenced by cow-level and cycle-level factors, including baseline activity, parity, milk production, health status, social behavior, and estrus intensity [2,5,21,40]. In agreement with this concept, both baseline-standardized and baseline-difference VeDBA and Gyro_mag showed clearer phase-related patterns than the corresponding SVM_acc variables. Baseline-standardized features described deviations from the normal phase in standard deviation units, whereas baseline-difference features preserved the original measurement scale and described the absolute change from baseline. These findings indicate that cow-specific baseline adjustment helped distinguish estrus-associated changes from normal inter-cow variability and support within-cow baseline adjustment as a biologically meaningful preprocessing step for future estrus-monitoring models [41,42].

The exploratory Combined Activity Index provided preliminary evidence for the value of integrating dynamic body acceleration and rotational movement into a single composite indicator. In practical terms, higher index values reflected greater combined locomotor activity and rotational head–neck movement. The elevation of this index during pre-estrus and standing estrus suggests increased behavioral arousal, restlessness, locomotor activity, and social or mounting-related interactions during these phases [2,5,43]. The VeDBA40 weighting was selected as a balanced compromise because it retained meaningful acceleration-derived information while giving slightly greater emphasis to gyroscope-derived movement, which showed a clear phase-related response in the present dataset. Nevertheless, this weighting was selected descriptively and was not optimized using predictive modelling or validated in an independent population. Therefore, the Combined Activity Index should be regarded as a candidate composite feature for future estrus-monitoring model development rather than as a validated estrus-detection tool.

### 4.2. Posture-Related Behavioral Changes During Natural Estrus

Posture-related behaviors complemented IMU-derived activity features by showing how natural estrus altered posture allocation, locomotion, and resting structure. The decrease in lying proportion during standing estrus is consistent with previous studies reporting reduced resting behavior and increased behavioral restlessness during estrus [2,5,11]. In parallel, the increased standing proportion during pre-estrus and standing estrus supports previous observations that cows spend more time in an upright and behaviorally active state during estrus expression, particularly because standing to be mounted is recognized as the primary behavioral sign of estrus [16,17]. Walking proportion also increased most clearly during standing estrus, agreeing with previous reports that estrus is associated with increased locomotor activity and movement-based activity alerts [1,2,11]. Unlike general activity-monitoring studies, the present study evaluated walking as a posture-related behavioral measure across biologically defined estrus phases, allowing more direct interpretation of the locomotor component of standing estrus. Therefore, walking serves as a complementary behavioral indicator to IMU-derived activity features, while its interpretation should consider housing space, social context, and individual cow variability [21,40].

Posture-related responses should be interpreted in relation to the environmental and social context of the study. Although reduced lying and increased standing and walking during standing estrus are consistent with estrus-associated restlessness and locomotor activity, these behaviors can also be influenced by housing conditions, available space, floor surface, herd size, social hierarchy, and the number of sexually active herd mates [2,16,40,44]. Therefore, the posture-related changes observed in this loose-barn setting should be interpreted cautiously, and future validation across different housing systems, herd sizes, and social group compositions is required to confirm their robustness as estrus-monitoring indicators.

The lying bout findings further clarified the structure of resting behavior during natural estrus. Previous studies have reported that cows reduce resting behavior and increase activity, restlessness, and locomotion during the estrus period [2,5,19]. Consistent with these reports, lying proportion decreased during standing estrus in the present study. However, lying bout rate did not differ significantly across phases, whereas mean lying bout duration was shorter during standing estrus. This pattern indicates that the reduction in lying behavior was related mainly to shorter and less sustained resting periods rather than to more frequent lying episodes [34,35]. Therefore, mean lying bout duration complements lying proportion by describing the temporal organization of resting behavior and providing a more detailed interpretation of resting disruption during standing estrus [36,37].

### 4.3. Biological and Practical Implications of the Phase-Based Framework

Previous studies have used different reference standards or criteria to define estrus, resulting in varying levels of biological and temporal resolution. Studies using activity sensors, visual estrus observation, or activity-monitor alerts have shown that estrus is associated with increased activity, reduced inactivity or rumination, mounting-related behavior, and a biological relationship with ovulation timing [5,11,20]. However, most sensor-based studies have focused on broad estrus versus non-estrus comparisons, general activity increases around estrus, or ovulation-related outcomes rather than the temporal progression of natural estrus across distinct behavioral phases [2,7]. The present study addressed this limitation by evaluating IMU-derived activity features, posture-related behaviors, and lying bout characteristics across biologically defined phases of natural estrus. Natural estrus was divided into six ovulation-anchored phases including normal, pre-estrus, standing estrus, late estrus, peri-ovulation, and early post-ovulation phases, using video-derived behavioral signs and ultrasound-confirmed ovulation. This phase-based framework enabled behavioral changes to be interpreted according to their temporal relationship to standing estrus and ovulation rather than as generalized increases in activity alone.

This phase-based approach showed that behavioral differentiation was most evident during standing estrus, when standing-to-be-mounted behavior was accompanied by increased dynamic movement, rotational activity, baseline-adjusted activity deviations, increased standing and walking proportions, and disruption of lying continuity. Walking proportion and the Combined Activity Index were significantly elevated during standing estrus compared with most non-standing phases, indicating that locomotor expression and integrated dynamic–rotational movement contributed to standing-estrus differentiation. These findings are consistent with previous studies identifying standing-to-be-mounted behavior as the primary behavioral sign of estrus and reporting increased mounting-related activity, locomotion, and behavioral restlessness during standing estrus [2,5,29]. Because standing estrus precedes ovulation, accurate identification of this phase is biologically and practically relevant for insemination-timing decisions [20,22]. Previous studies have reported that ovulation occurs approximately 24–33 h after estrus onset and 15–22 h after estrus end, although these intervals vary according to cow-level factors, estrus definition, observation frequency, and ovulation-monitoring method [44,45]. In the present study, the 6 h peri-ovulation window was estimated to begin approximately 29.18 h after the first observed standing-to-be-mounted event and 17.77 h after the last observed standing-to-be-mounted event, with the midpoint occurring approximately 32.14 h and 20.73 h after these respective reference points. These estimates are broadly consistent with previous reports of ovulation timing relative to estrus onset and estrus end [46,47]. Therefore, phase-specific differentiation of activity and posture-related features, particularly walking proportion and the Combined Activity Index during standing estrus, provides a biological basis for developing future models to estimate the ovulation window and support insemination-timing decisions, although this application requires validation in larger datasets with direct evaluation of insemination outcomes.

### 4.4. Strengths and Limitations

Several strengths and limitations should be considered when interpreting these findings. A major strength was the use of naturally occurring estrus under routine farm conditions, allowing behavioral expression to be evaluated without hormonal synchronization. In addition, the combination of continuous video observation and ultrasound-confirmed ovulation strengthened the biological reference standard for phase classification [5,22].

A key limitation of the present study was the small biological sample included in the final analysis. Although the dataset contained a large number of 10 s sensor observations, these records should not be interpreted as independent biological replicates because they were derived from five cows and eleven natural estrus cycles from a single loose-barn facility. In addition, because some cows contributed multiple estrus cycles, the repeated structure of cycles within cows should be considered when interpreting the strength of inference. Given the limited number of cows and cycles available for estimating random-effect variance components, the present study should be interpreted as an exploratory characterization of estrus-associated behavioral dynamics rather than as a source of definitive population-level estimates. Estrus expression can vary according to cow-level factors, cycle-level variation, herd size, housing environment, social interaction, health status, and management conditions [2,5,40,44]. Consequently, the generalizability of the observed IMU-derived activity and posture-related patterns to other farms, breeds, housing systems, management conditions, and sensor placements remains constrained.

Another methodological limitation was the 10 s averaging interval of the IMU records, which resulted in an effective sampling frequency of 0.1 Hz. Although this resolution was suitable for summarizing broad phase-level activity and posture-related patterns, it was not designed for high-precision detection of brief estrus-related events. Short-duration behaviors, such as individual mounting attempts, brief standing-to-be-mounted events, or rapid head–neck movements, could have been attenuated or incompletely represented. Therefore, the IMU-derived features should be interpreted as indicators of broader phase-level movement patterns rather than event-level behavioral detections. Future studies using higher-frequency raw IMU data, larger cow populations, more estrus cycles, independent datasets, and multi-farm validation are required to evaluate event-level estrus behavior detection, confirm the robustness of these candidate features, and support stronger population-level inference [23,24].

## 5. Conclusions

This exploratory study showed that IMU-derived activity features and posture-related behaviors varied across biologically defined phases of natural estrus in dairy cows, with the clearest differentiation occurring during pre-estrus and standing estrus. VeDBA and Gyro_mag were more informative than SVM_acc for characterizing estrus-related movement changes, while baseline-adjusted variables improved interpretation by expressing deviations relative to each cow’s normal activity level. Posture-related variables complemented activity features, as standing estrus was characterized by reduced lying proportion, increased standing and walking proportions, and shorter mean lying bout duration. The Combined Activity Index integrating VeDBA and Gyro_mag showed potential as a candidate composite feature of estrus-related activity, although further validation is required. Overall, these findings support the use of an ovulation-anchored six-phase framework to characterize phase-specific behavioral changes during natural estrus and identify candidate variables for future estrus-monitoring models. Validation in larger, independent, and more diverse dairy cow datasets is required before practical application for standing-estrus differentiation, ovulation-window estimation, or insemination-timing support.

## Figures and Tables

**Table 1:** Definition of biologically defined estrus phases used for phase-level analysis.

Phase	Phase Name	Meaning
1	Normal phase	Baseline period before the onset of estrus-associated behavioral changes. This phase extended from 72 h before the ovulation midpoint to the last observation before the beginning of pre-estrus.
2	Pre-estrus	Period beginning at the first valid secondary estrous sign, defined as an observation window with an estrus score ≥15 and <100, followed by another secondary estrous sign within 2 h. This phase ended at the last observation before the first standing estrus event.
3	Standing estrus	Period from the first to the last observation window in which the cow exhibited standing-to-be-mounted behavior or had an estrus score ≥100.
4	Late estrus	Period beginning immediately after the cessation of standing estrus behavior and continuing until the last observation before the peri-ovulatory period.
5	Peri-ovulation	Six-hour ultrasound-confirmed ovulation window, defined as the interval between the last monitoring time point at which the Graafian follicle was observed and the first monitoring time point at which it was no longer visible. This phase represented the biologically confirmed period covering ovulation.
*	Ovulation midpoint	Designated ovulation time, calculated as the midpoint of the peri-ovulation period.
6	Early post-ovulation	Period beginning immediately after the end of the peri-ovulation window and continuing until 24 h after the ovulation midpoint. As the ovulation midpoint was designated as the middle of the 6 h peri-ovulation window, this phase comprised the subsequent 21 h observation period after peri-ovulation.

Footnote: The asterisk (*) refers to the ovulation midpoint, which represents the operationally designated ovulation time, calculated as the midpoint of the 6 h ultrasound-confirmed peri-ovulation window.

**Table 2:** Activity and postural features used for phase-level analysis.

Feature	Type	Description
SVM_acc	Activity	Overall linear movement intensity
VeDBA	Activity	Dynamic body movement (activity proxy)
Gyro_mag	Activity	Summarized rotational activity
Base_z_ SVM_acc	Activity	Baseline-standardized linear movement intensity
Base_z_ VeDBA	Activity	Baseline-standardized dynamic body movement
Base_z_ Gyro_mag	Activity	Baseline-standardized rotational activity
Percent Lying	Posture	Proportional resting behavior
Percent Standing	Posture	Proportional standing behavior
Percent Walking	Posture	Proportional locomotor activity
Lying bout rate	Posture	Frequency of lying episodes
Mean lying bout duration	Posture	Average duration per lying episode

**Table 3:** Definition of cow behaviors used for behavioral annotation.

Behavior	Definition
Lying	The cow was in a resting posture with the ventral body surface in contact with the ground, supported by the sternum and one or both thighs. The neck was positioned vertically or horizontally and could be flexed backward toward the hindquarters. Lateral recumbency, in which the cow lay fully on its side, was excluded to maintain consistency in posture-based labeling.
Standing	The cow remained upright, supported by at least three legs, without forward or backward movement. The neck was aligned along the vertical axis, although minor movements related to comfort or social interactions could occur.
Walking	The cow showed progressive forward or backward movement covering more than two feet. The behavior involved sequential limb movements, with the head generally held in an upright position.

**Table 4:** Data structure and distribution of observations across estrus phases.

No. of Cycles	Estrus Phase Observations (*n*)						Total Observations (*n*)	Cow ID
	1	2	3	4	5	6		
1	13,295	696	4015	3308	1524	6360	29,198	1
2	6545	3727	1145	8405	1180	4281	25,283	1
3	9072	2139	5220	4479	1836	9318	32,064	1
4	6485	1044	2824	4738	1530	6958	23,579	2
5	11,835	1412	6073	3647	2022	6466	31,455	2
6	5710	2210	2540	4655	1566	1620	18,301	2
7	3057	459	564	1015	628	1526	7249	3
8	2332	2883	6580	2768	1986	6961	23,510	3
9	6819	3620	1464	9270	2039	7100	30,312	3
10	10,589	3370	1078	7940	2003	6872	31,852	4
11	12,361	2322	4284	5192	1995	6380	32,534	5
Total	88,100	23,882	35,787	55,417	18,309	63,842	285,337	
	30.9%	8.4%	12.5%	19.4%	6.4%	22.4%	100.0%	

Footnote: Values indicate the number of 10 s IMU observations assigned to each estrus phase. Percentages reflect the proportion of total observations in each phase.

## Data Availability

The data and model were not deposited in an official repository. Data are available upon request to the corresponding author.

## Abbreviations

The following abbreviations are used in this manuscript:

AI	Artificial insemination
ANOVA	Analysis of variance
Base_z	Baseline-standardized value
Base_diff	Baseline-difference value
CCTV	Closed-circuit television
DBA	Dynamic body acceleration
Gyro_mag	Gyroscope magnitude
IMU	Inertial measurement unit
SD	Standard deviation
SVM_acc	Signal vector magnitude of acceleration
VeDBA	Vectorial dynamic body acceleration

## Appendix A. Estrus Phase Duration Characteristics

**Table A1:** Overall phase duration summary (h) across 5 cows and 11 estrous cycles.

Phase	Description	Mean ± SD (h)	Median (h)	Min–Max (h)
1	Normal phase	26.92 ± 7.41	26.31	14.50–36.97
2	Pre-estrus	8.74 ± 6.69	6.87	2.06–26.80
3	Standing estrus	11.41 ± 5.85	12.64	3.19–19.45
4	Late estrus	17.77 ± 8.56	15.92	8.23–34.01
5	Peri-ovulation	5.92 ± 0.24	6.00	5.18–6.00
6	Early post-ovulation	20.05 ± 1.92	21.00	14.91–21.00

**Table A2:** Summary of activity, posture, and lying bout variables across estrus phases.

Type	Features	Phase					
		1	2	3	4	5	6
Absolute activity	SVM_acc	0.30 ± 0.04 ^a^	0.33 ± 0.08 ^a^	0.32 ± 0.05 ^a^	0.32 ± 0.04 ^a^	0.32 ± 0.06 ^a^	0.33 ± 0.05 ^a^
	VeDBA	0.11 ± 0.02 ^b^	0.14 ± 0.06 ^ab^	0.17 ± 0.05 ^a^	0.12 ± 0.02 ^b^	0.10 ± 0.03 ^b^	0.12 ± 0.02 ^b^
	Gyro_mag	11.12 ± 4.15 ^c^	20.19 ± 6.56 ^ab^	22.10 ± 6.85 ^a^	14.43 ± 3.87 ^bc^	10.05 ± 6.75 ^c^	11.83 ± 1.95 ^c^
Baseline-Standardized	SVM_acc	-	0.19 ± 0.45 ^a^	0.12 ± 0.33 ^a^	0.11 ± 0.32 ^a^	0.14 ± 0.28 ^a^	0.18 ± 0.40 ^a^
	VeDBA	-	0.18 ± 0.39 ^abc^	0.46 ± 0.30 ^a^	0.08 ± 0.14 ^b^	−0.10 ± 0.30 ^c^	0.05 ± 0.12 ^b^
	Gyro_mag	-	0.96 ± 0.50 ^a^	1.14 ± 0.44 ^a^	0.37 ± 0.50 ^b^	−0.11 ± 0.40 ^b^	0.12 ± 0.33 ^b^
Baseline-Difference	SVM_acc	-	0.04 ± 0.08 ^a^	0.02 ± 0.06 ^a^	0.02 ± 0.06 ^a^	0.03 ± 0.05 ^a^	0.03 ± 0.06 ^a^
	VeDBA	-	0.03 ± 0.05 ^abc^	0.06 ± 0.04 ^b^	0.01 ± 0.02 ^a^	−0.02 ± 0.04 ^c^	0.01 ± 0.02 ^ac^
	Gyro_mag	-	9.08 ± 4.44 ^a^	10.98 ± 4.20 ^a^	3.32 ± 4.75 ^b^	−1.07 ± 5.21 ^b^	0.71 ± 3.30 ^b^
Combined Activity Index	VeDBA40:Gyro60	15.79 ± 3.20 ^b^	27.41 ± 7.34 ^a^	30.36 ± 5.33 ^a^	20.57 ± 4.87 ^b^	14.09 ±6.93 ^b^	17.30 ±3.12 ^b^
Posture proportion (%)	Percent Lying	60.59 ± 5.73 ^c^	28.29 ± 21.37 ^ab^	13.89 ± 9.71 ^a^	53.23 ± 8.89 ^bc^	68.31 ± 24.94 ^c^	54.13 ± 8.65 ^bc^
	Percent Standing	31.87 ± 5.30 ^b^	58.06 ± 23.27 ^a^	70.16 ± 14.74 ^a^	36.00 ± 8.83 ^b^	26.19 ± 16.14 ^b^	39.62 ± 7.96 ^b^
	Percent Walking	1.98 ± 0.72 ^c^	6.35 ± 8.67 ^abc^	13.70 ± 6.95 ^b^	2.54 ± 1.30 ^ac^	1.17 ± 1.15 ^a^	1.46 ± 1.04 ^ac^
Lying bout characteristic (min)	Lying Bout Rate	0.61 ± 0.19 ^a^	0.66 ± 0.68 ^a^	0.91 ± 1.21 ^a^	0.58 ± 0.16 ^a^	0.69 ± 0.49 ^a^	0.75 ± 0.36 ^a^
	Mean Lying Bout Duration	64.37 ± 21.34 ^bc^	30.60 ± 22.22 ^ab^	22.33 ± 29.77 ^a^	62.98 ± 25.34 ^bc^	76.00 ± 31.08 ^c^	52.08 ± 21.44 ^bc^

Footnote: Values are presented as mean ± SD. Different superscript letters indicate statistically significant differences between phases (*p* < 0.05).
